# Fatty Acids and Stable Isotope Ratios in Shiitake Mushrooms (*Lentinula edodes*) Indicate the Origin of the Cultivation Substrate Used: A Preliminary Case Study in Korea

**DOI:** 10.3390/foods9091210

**Published:** 2020-09-01

**Authors:** Ill-Min Chung, So-Yeon Kim, Jae-Gu Han, Won-Sik Kong, Mun Yhung Jung, Seung-Hyun Kim

**Affiliations:** 1 Department of Crop Science, College of Sanghuh Life Science, Konkuk University, Seoul 05029, Korea; imcim@konkuk.ac.kr (I.-M.C.); hellosy1@konkuk.ac.kr (S.-Y.K.); 2 National Institute of Horticultural and Herbal Science, Rural Development Administration, Eumseong 27709, Korea; hanjaegu@korea.kr (J.-G.H.); wskong@korea.kr (W.-S.K.); 3 Department of Food Science and Biotechnology, Graduate School, Woosuk University, Wanju-gun 55338, Korea; munjung@woosuk.ac.kr

**Keywords:** shiitake mushroom, cultivation substrate, harvesting cycle, fatty acid, stable isotope ratio

## Abstract

Shiitake mushroom (*Lentinula edodes*) is commonly consumed worldwide and is cultivated in many farms in Korea using Chinese substrates owing to a lack of knowledge on how to prepare sawdust-based substrate blocks (bag cultivation). Consequently, issues related to the origin of the Korean or Chinese substrate used in shiitake mushrooms produced using bag cultivation have been reported. Here, we investigated differences in fatty acids (FAs) and stable isotope ratios (SIRs) in shiitake mushrooms cultivated using Korean and Chinese substrates under similar conditions (strain, temperature, humidity, etc.) and depending on the harvesting cycle. The total FA level decreased significantly by 5.49 mg∙g^−1^ as the harvesting cycle increased (*p* < 0.0001); however, no differences were found in FAs between shiitake mushrooms cultivated using Korean and Chinese substrates. Linoleic acid was the most abundant FA, accounting for 77–81% of the total FAs during four harvesting cycles. Moreover, the SIRs differed significantly between the Korean and Chinese substrates, and the harvesting cycles resulted in smaller maximum differences in SIR values compared to those of the cultivation substrate origins. Our findings contribute to the identification of the geographical origin of shiitake mushrooms and may have potential applications in international shiitake-mushroom markets.

## 1. Introduction

Shiitake mushroom (*Lentinula edodes*) is globally the second most commonly cultivated mushroom owing to its unique taste and flavor [1]. It accounts for approximately 22% of the total global mushroom production, second to *Agaricus bisporus*. Shiitake mushrooms are rich in nutrients such as essential amino acids, vitamins (B_1_, B_2_, C, and D), minerals, dietary fibers, and β-glucan [2,3,4,5]. Compared to other widely cultivated mushrooms, shiitake mushrooms contain higher levels of macronutrients (except for proteins), sugars, tocopherols, and polyunsaturated fatty acids (FAs) but lower levels of saturated FAs [6]. Furthermore, shiitake mushrooms possess anticancer, antitumor, antimicrobial, anti-inflammatory, and hypercholesterolemic activities [2,7,8].

*Lentinula* spp. are saprophytic wood-rotting fungi; they usually grow on the cambium of dried logs by absorbing nutrients from wood breakdown. Traditionally, these mushrooms were cultivated in Asia by inoculating wood logs under favorable outdoor conditions in the following manner: select a proper tree (40–45% moisture; usually of the family *Fagaceae*); prepare spawn; inoculate the spawn into logs; place them under proper conditions for the development of mycelium; and harvest for 3–4 years once the mycelia grow well and generate good fruiting bodies. However, owing to the limited supply of appropriate hardwood, the specific cultivation conditions, and the long time required for traditional shiitake cultivation using wood logs, the bag cultivation method (a synthetic log system) is now widely used to consistently produce high-quality mushrooms [1]. Oak sawdust is the most popular basal ingredient used (approximately 50% of the time) to generate synthetic logs or cultivation substrates for bag cultivation. Other components, such as straw (rice and wheat), corn cobs, or mixtures of these components, are also commonly used in different proportions according to country, region, or farm. Moreover, various starch-based supplements (i.e., wheat bran, rice bran, millet, rye, and maize powder) can serve as additional nutrients to prepare optimum growth substrates [9], which could improve the quality, productivity, flavor, and storage of the cultivated mushrooms [7,10,11]. For example, substrates composed of gypsum, manure, cottonseed hulls, corn cobs, or wheat straw are used for cultivating mushrooms in the USA; using these substrates, mushrooms have been harvested four or five times from the same bag [12].

The global mushroom production has significantly increased from 2.68 to 10.8 million tons within the past two decades. China is the largest producer, contributing to 72% of the world’s total mushroom production. Shiitake mushrooms (25%) are the most commonly cultivated mushrooms in China, followed by *Pleurotus ostreatus* (15%) and *A. bisporus* (9.3%) [13].

Shiitake mushrooms are commonly consumed in East Asian countries, including China, Korea, and Japan [14]. Despite the increasing consumption of shiitake mushrooms in Korea, techniques for preparing synthetic logs for bag cultivation are still not standardized; therefore, many novice shiitake mushroom farms have continued to use either spawn-inoculated substrate or substrates imported from China [15]. Consequently, origin labeling for shiitake mushrooms cultivated in Korea using Chinese substrates is becoming a major issue for consumers and it is often unclear whether the mushrooms have been produced using Korean or Chinese substrates. Moreover, origin labeling based on cultivation substrate depends on substrate preparation duration, inoculation, and cultivation in a specific country. For example, if shiitake mushrooms are cultivated using Chinese substrate and grown for a longer time in Korea than that followed in China, the mushrooms are labeled as produced in Korea and not China.

However, to the best of our knowledge, few studies have characterized shiitake mushrooms cultivated in Korea using Chinese and domestic substrate. Therefore, in this study, we aimed to examine and compare FA levels and stable isotope ratios (SIRs) in shiitake mushrooms cultivated using two substrates depending on the harvesting cycle. Our preliminary findings will enhance the knowledge regarding FA levels and SIRs in shiitake mushrooms cultivated in Korea using different substrates.

## 2. Materials and Methods

### 2.1. Shitake Mushroom Collection

Shiitake mushrooms (cultivar L808) cultivated using Korean and Chinese substrates were collected from two farms (A and B) located in Jangsu-gun, Korea (35°33′ N 127°29′ E), in 2019. In fact, the components of the Korean and Chinese substrates used in this study were not provided clearly from shiitake farms. However, in general, the Korean substrate comprised approximately 80% oak tree sawdust and 20% rice (or wheat) bran, with a water content of 55–60%. Components such as straw, corn cobs, or additional supplements (i.e., gypsum and calcium carbonate) are sometimes used for improving shiitake quality or yield in Korea [16,17,18]. In contrast, Chinese substrates were usually made of (aged broadleaf) sawdust or easily accessible any agro-industrial organic material. Recently a typical substrate formulation for shiitake cultivation in China is composed of cottonseed hull (50%), sawdust (28%), wheat bran (20%), gypsum (1%), and sugar (1%) [19]. During shiitake cultivation using these substrates, the temperature and humidity in the greenhouse system are maintained at 20–25 °C and ~70%, respectively, during substrate culture, and air is circulated to control humidity in the greenhouse. Furthermore, the temperature and humidity in the greenhouse system are maintained at 15–23 °C and 80–90%, respectively, on an average, during mushroom growth [18].

Next, approximately 1 kg (~100 fruiting bodies) shiitake mushrooms were randomly harvested in four cycles for each substrate from the two farms. At each harvesting cycle, the collected mushrooms (1 kg) were immediately transferred to the laboratory within a day, 30 fruiting bodies with a 7–8 cm diameter were chosen, and the mushrooms were then lyophilized below −70 °C for 3 days (Freezone 4.5; Labconco, MO, USA). Afterward, five lyophilized fruit bodies were pulverized into a fine powder (≤400 μm) using a grinder and used as a replicate. Thus, five replicates (*n* = 5) were prepared for each harvesting cycle per cultivation substrate from each farm. All samples were then stored in a desiccator at room temperature (~24 °C; humidity, <20%) for sample preparation for FA and SIR analysis. When sample preparation needed a longer time (>7 days), the mushrooms were tightly sealed and frozen (−75 °C) until reuse. These samples were used for FA-level and SIR analyses after re-lyophilization.

### 2.2. Chemicals and Reagents

Isooctane, benzene, heptane, and methanol were purchased from Fisher Scientific Korea Ltd. (Seoul, Korea) or Junsei Chemical Ltd. (Tokyo, Japan). Standards of a 37-fatty acid methyl ester (FAME) mixture (CRM47885), pentadecanoic acid (C15:0, P6125), sulfuric acid (H_2_SO_4_), dimethyl sulfoxide, 2,2-dimethoxypropane (DMP), and dichloromethane were purchased from Sigma-Aldrich Co. (St. Louis, MO, USA).

### 2.3. FA Extraction, Derivatization, and Analysis

FAs in the mushroom samples were converted to FAMEs via transesterification. Briefly, mushroom powder (30 mg) and internal standard (C15:0; 0.2 mg) were transferred to amber vials. A methylation mixture (680 μL) comprising methanol/benzene/DMP/sulfuric acid (19.5:10:2.5:1, by volume) and heptane (400 μL) were added to the vials. The vials were then tightly capped with Teflon-lined caps and gently shaken in a water bath (BioFree Co., Ltd., Cheongju-Si, Korea) at 80 °C for 2 h. The samples were then cooled to room temperature, and supernatants containing FAMEs were transferred into a microcentrifuge tube and centrifuged for 1 min. Lastly, the supernatants were transferred to amber vials using a 300 μL insert [20,21].

After the conversion of FAs to FAMEs, the FA composition and levels in the shiitake mushroom samples were measured using gas chromatography coupled with a flame ionization detector (GC-FID) system (Agilent Co., Ltd., Santa Clara, CA, USA). An HP-INNOWAX capillary column (19091N; 0.25 mm × 30 m, 0.25 μm; Agilent Co., Ltd.) was used to separate FAMEs. The injection volume was 1 μL, with a 1:50 split mode. The carrier gas was helium (10 mL·min^−1^), and the FID flame gas was composed of hydrogen (35 mL·min^−1^) and air (300 mL·min^−1^). The GC oven temperature was initially set at 100 °C held for 2 min, increased to 150 °C at a rate of 5 °C·min^−1^, held for 2 min, and then finally increased to 240 °C at a rate of 5 °C min^−1^ held for 5 min. The inlet and FID temperatures were set at 230 °C and 250 °C, respectively, and the total analytical time was 64 min/sample [20].

### 2.4. Quantification of FAs

**Figure 1:**
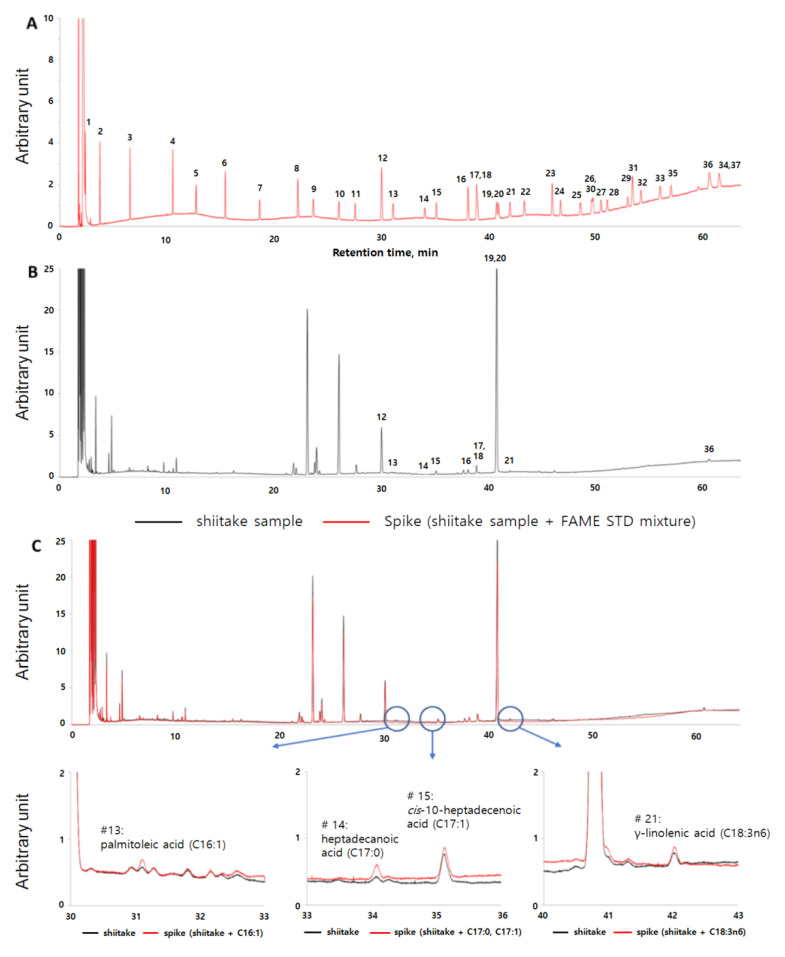
Gas chromatography coupled with a flame ionization detector (GC-FID) chromatograms of fatty acids (FAs) in shiitake mushrooms. (**A**) Chromatograms of the 37-FAME standards. (**B**) First harvested shiitake mushrooms. (**C**) Spike test (C16:0, C17:0, C17:1, C18:3 *n*-6). 1: butyric acid (C4:0), 2: caproic acid (C6:0), 3: caprylic acid (C8:0), 4: capric acid (C10:0), 5: undecanoic acid (C11:0), 6: lauric acid (C12:0), 7: tridecanoic acid (C13:0), 8: myristic acid (C14:0), 9: myristoleic acid (C14:1), 10: pentadecanoic acid (C15:0), 11: *cis*-10-pentadecenoic acid (C15:1), 12: palmitic acid (C16:0), 13: palmitoleic acid (C16:1), 14: heptadecanoic acid (C17:0), 15: *cis*-10-heptadecenoic acid (C17:1), 16: stearic acid (C18:0), 17, 18: oleic (*cis*) and elaidic (*trans*) acid (C18:1 *n*-9), 19, 20: linoleic (*cis*) and linolelaidic (*trans*) acid (C18:2 *n*-6), 21: γ-linolenic acid (C18:3 *n*-6), 22: α-linolenic acid (C18:3 *n*-3), 23: arachidic acid (C20:0), 24: *cis*-11-eicosenoic acid (C20:1 *n*-9), 25: *cis*-11,14-eicosadienoic acid (C20:2), 26, 30: *cis*-8,11,14-eicosatrienoic (C20:3 *n*-6) and heneicosanoic acid (C21:0), 27: *cis*-11,14,17-eicosatrienoic acid (C20:3 *n*-3), 28: arachidonic acid (C20:4 *n*-6), 29: *cis*-5,8,11,14,17-eicosapentaenoic acid (C20:5 *n*-3), 31: behenic acid (C22:0), 32: erucic acid (C22:1 *n*-9), 33: *cis*-13,16-docosadienoic acid (C22:2), 35: tricosanoic acid (C23:0), 36: lignoceric acid (C24:0), and 34, 37: *cis*-4,7,10,13,16,19-docosahexaenoic acid (C22:6 *n*-3) and nervonic acid (C24:1 *n*-9).

For quantification, the 37-FAME standard (STD) mixture (1 mL) was dissolved in 9 mL dichloromethane to prepare a stock solution. Each FA in the shiitake mushroom sample was identified by comparing the retention time with that of the properly diluted FAME STD mixture. Furthermore, the FAME STD mixture was added to the sample aliquots to confirm the accuracy of the FA peak assignment in the samples of interest. Finally, the FA level (mg∙g^−1^, dry basis) was calculated using the method specified in the Korean Food and Drug Administration Food Code, considering the response and conversion factor for each FA [22]. Figure 1 shows representative GC-FID chromatograms of the 37-FAME STDs and shiitake mushrooms harvested during the first cycle (farm A, Chinese substrate). The correct FA peak assignment (Figure 1C) was confirmed via the spike test performed using the 37-FAME STD mixture including palmitoleic acid (C16:1), heptadecanoic acid (C17:0), *cis*-10-heptadecenoic acid (C17:1), and γ-linolenic acid (C18:3 *n*-6).

### 2.5. Analysis of C, N, O, and S SIRs

The pulverized shiitake powder was transferred to and encapsulated in a tin capsule (3.5 mm × 17 mm; IVA Analysentechinik e. K., Dusseldorf, Germany) for δ^13^C and δ^15^N, another tin capsule (9 mm × 10 mm) for δ^34^S, and a silver capsule (3.5 mm × 5.0 mm; Elemental Microanalysis, Okehampton, UK) for δ^18^O. For reliable SIR measurements, 2.5 mg shiitake powder was used for simultaneous δ^13^C and δ^15^N analysis, approximately 0.2 mg was used for δ^18^O analysis, and approximately 20 mg was used for δ^34^S analysis. The encapsulated samples were placed in a desiccator until C, N, O, and S SIRs were measured [23].

The C, N, O, and S SIRs were measured using an isotope ratio mass spectrometer (IRMS) coupled with an elemental analyzer system, as previously described [24]. Briefly, after combustion or thermal decomposition, the shiitake samples were converted to CO_2_ and N_2_ for δ^13^C and δ^15^N measurements, respectively, CO for δ^18^O measurement, and SO_2_ for δ^34^S analysis. The samples were then evaluated using the IRMS system. For reliable measurements, such as scale normalization, correction of potential measurement variation, and quality assurance, certain laboratory reference materials (RMs), which were compositionally similar to the samples, were also analyzed using the same IRMS system.

The final C, N, O, and S SIRs were reported in parts per thousand (per mill, ‰), compared with values of internationally established reference standards using the following formula: δ, ‰ = ([R_unknown_ − R_standard_]/R_standard_), where R is the C, N, O, or S SIR (e.g., ^13^C/^12^C, ^15^N/^14^N, ^18^O/^16^O, ^34^S/^32^S, respectively) of the shiitake mushroom samples and the international reference standards. Vienna PeeDee Belemnite, atmospheric N_2_, Vienna Standard Mean Ocean Water, and Vienna Canyon Diablo Troilite were used as the international reference standards for C, N, O, and S SIRs, respectively [25]. In this study, the mean absolute accuracy for calibrated RMs was ±0.03‰ for δ^13^C, ±0.04‰ for δ^15^N, ±0.17‰ for δ^18^O, and ±0.29‰ for δ^34^S IRMS measurements.

### 2.6. Statistical Analysis

Results have been reported as mean (± standard deviation), and all data were analyzed using SAS software (version 9.4; SAS Institute Inc., Cary, NC, USA) with the general linear model. Least significant difference (LSD) tests with a probability level of 0.05 were used to determine differences among samples based on either the harvesting cycle or substrate origin. Furthermore, statistical analysis was conducted using two-way ANOVA with *p*-values in SAS to evaluate the effects of the main factor and its interactions.

## 3. Results and Discussion

### 3.1. Comparison of FAs in Shiitake Mushrooms Based on the Harvesting Cycle and Substrate Origin

**Figure 2:**
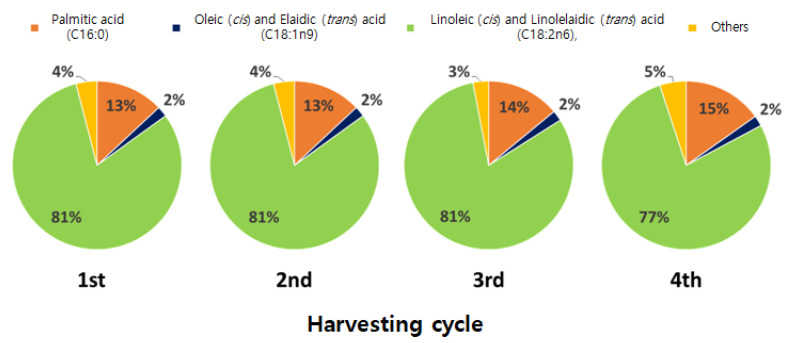
Changes in the FA levels in shiitake mushrooms based on the harvesting cycle. The results indicate the mean value of the samples produced using both Korean and Chinese substrates.

Table 1 summarizes the FA composition and levels in shiitake mushrooms based on the harvesting cycle (H) and substrate origin (S) and also shows the associated two-way ANOVA *p*-value for the main factor (H, S) and its interactions (H × S). Eight FAs—C16:0, C17:0, C17:1, C18:0, C18:1 *n*-9, C18:2 *n*-6, C18:3 *n*-6, and C24:0 were found in shiitake mushrooms; however, C17:0 and C18:3n-6 were observed at below the limit of detection (LOD) based on the signal-to-noise ratio (S/N = 3). Additionally, the level of total FAs decreased significantly by 5.49 mg∙g^−1^ as the harvesting cycle increased (*p* < 0.0001; Table 1). Unsaturated FAs (UFAs) and saturated FAs (SFAs) accounted for approximately 83 and 16% of the total FAs, respectively, and UFAs showed a particularly significant decrease as the harvesting cycle increased (Table 1). Of note, C18:2n-6 linoleic acid was the most abundant FA, accounting for 77–81% of the total FAs in shiitake mushrooms during the four harvesting cycles (Figure 2). These findings are similar to those of a prior study that reported that shiitake mushrooms harbored the highest levels of polyunsaturated FAs (PUFAs) and the lowest levels of SFAs among all mushrooms [6].

**Figure 3:**
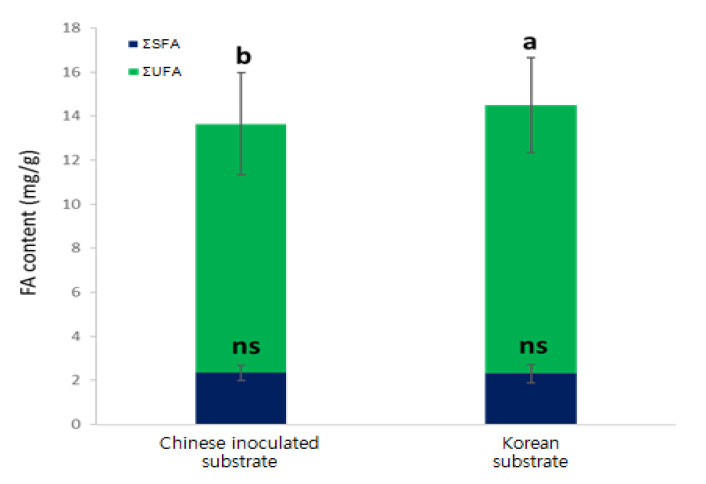
Comparison of the total FA composition of shiitake mushrooms from two cultivation substrates. ΣSFA: sum of saturated FAs (SFAs) and ΣUFA: sum of unsaturated FAs (UFAs). a and b Values with different superscripts differ significantly with respect to the substrate origin. ns: non-significant.

**Figure 4:**
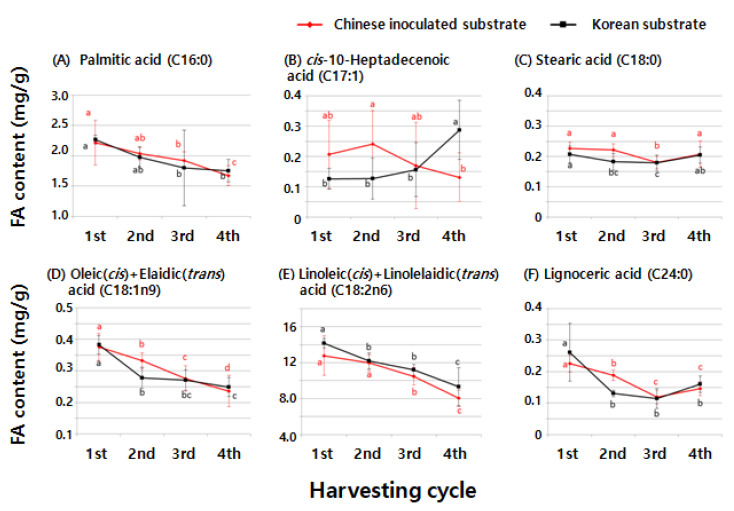
Comparison of the FA levels in shiitake mushrooms cultivated using Chinese and Korean substrates according to the harvesting cycle. (**A**) Palmitic acid (C16:0), 18: 19, 20: (**B**) *cis*-10-heptadecenoic acid (C17:1), (**C**) stearic acid (C18:0), (**D**) oleic (*cis*) and elaidic (*trans*) acid (C18:1 *n*-9), (**E**) linoleic (*cis*) and linolelaidic (*trans*) acid (C18:2 *n*-6), and (**F**) lignoceric acid (C24:0). a–c Values with different superscripts differ significantly with respect to the harvesting cycles of shiitake produced by each substrate.

**Figure 5:**
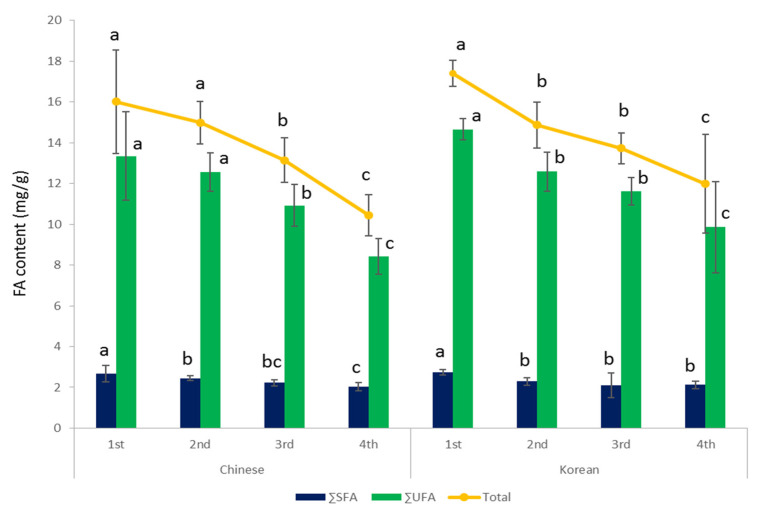
Comparison of FA compositions and levels in shiitake mushrooms cultivated using Chinese or Korean substrates based on the harvesting cycle. ΣUFA: sum of UFAs, ΣSFA: sum of SFAs, Total: total amount of FAs measured in mushrooms. a–c Values with different superscripts differ significantly with respect to the harvesting cycles of shiitake produced by each substrate.

The total FA levels were approximately 6% higher in shiitake mushrooms cultivated using the Korean substrate than in those cultivated using the Chinese substrate (*p* < 0.05, Table 1). Interestingly, linoleic acid contents were also approximately 8% higher in shiitake mushrooms cultivated using the Korean substrate than in those cultivated using the Chinese substrate. In contrast, the SFA content did not differ based on the substrate origin (Table 1, Figure 3). The levels of most FAs found in shiitake mushrooms decreased as the harvesting cycles increased owing to their limited nutrient sources; however, the origin of the substrate did not affect FA composition or levels (Figure 4). Furthermore, shiitake mushrooms cultivated using Korean or Chinese substrates contained α-linoleic acid levels that were below the LOD (approximately 0.08 mg∙g^−1^). In addition, UFA and total FA levels continually decreased in the shiitake mushrooms cultivated using Korean or Chinese substrate until the fourth harvesting cycle, whereas SFA levels did not change much after the second harvesting cycle (Figure 5).

Acetyl CoA carboxylase controls the carbon flux for FA synthesis. Therefore, FA assembly depends on acyl carrier proteins via four reaction cycles, which elongate the acyl chain by two carbons (acetyl CoA) in each cycle [26]; desaturation is catalyzed by membrane-bound fatty acid desaturases [27]. Shiitake mushrooms are saprophytic wood-rotting fungi that grow by absorbing nutrients from wood breakdown. Thus, when sawdust is widely used as a substrate for bag cultivation, only limited amounts of carbon are available for acetyl CoA, which is essential for FA synthesis.

A prior study has shown that low-temperature shock treatment of the substrate alters the lipid profile and accelerates the initiation of sporophores in *L. edodes*, resulting in decreased palmitic acid but increased linoleic acid levels [28]. However, bag cultivation using oak sawdust is well controlled in response to certain environmental conditions, such as temperature, humidity, and air circulation, in greenhouse cultivation. Therefore, in this study, linoleic acid levels were found to have significantly decreased as the harvesting cycle increased, and this result may be explained by the limited carbon availability in the substrate during the late harvesting cycle. Additionally, the FA levels in shiitake mushrooms also vary according to the substrate origin; however, the reason is not clear owing to the limited sample size as well as non-analysis of substrate difference in this study. Thus, for describing the effect of the substrate on FA levels, more shiitake samples need to be analyzed together with the substrates. Because essential FAs such as linoleic and α-linolenic acids are involved in controlling in vivo inflammation via prostaglandin synthesis, larger amounts of PUFAs are highly desirable, and foods with a PUFA/SFA ratio of <0.45 are considered less healthy [29,30,31]. In this study, shiitake mushrooms cultivated using both Korean and Chinese substrates showed higher PUFA/SFA ratios (>4); however, this ratio was decreased by approximately 16% as the harvesting cycle increased. Hence, at earlier harvesting cycles, shiitake mushrooms cultivated using the Korean substrate may contain more nutritionally desirable FAs, such as linoleic acid, compared to those cultivated using the Chinese substrate.

### 3.2. Comparison of δ^13^C, δ^15^N, δ^18^O, and δ^34^S in Shiitake Mushrooms Based on the Harvesting Cycle and Substrate Origin

**Figure 6:**
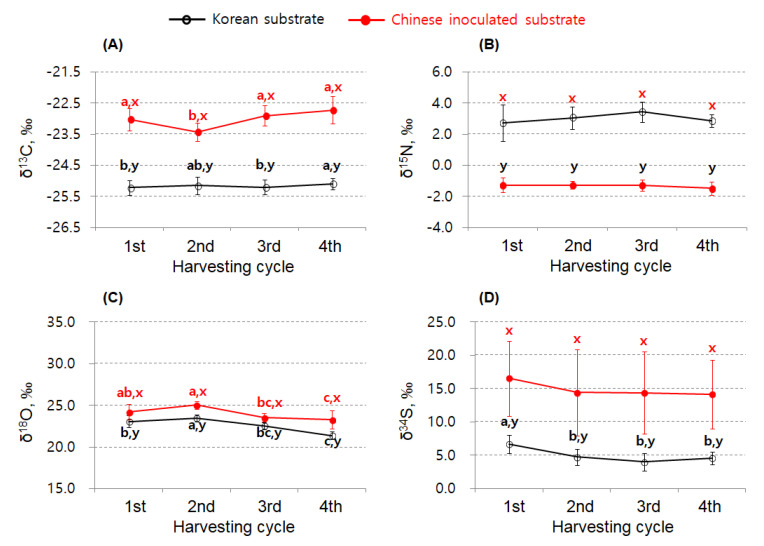
Comparison of δ^13^C, δ^15^N, δ^18^O, and δ^34^S variations based on the harvesting cycle in shiitake mushrooms cultivated using Korean or Chinese substrate. Values with different superscripts significantly differ with respect to the harvesting cycle (a–c) or substrate origin (x, y). The δ^13^C (**A**), δ^1^^5^N (**B**), δ^1^^8^O (**C**), and δ^3^^4^S (**D**) indicate the variations according to the harvesting cycle between Chinese inoculated and Korean substrate.

Figure 6 shows the differences in δ^13^C, δ^15^N, δ^18^O, and δ^34^S in shiitake mushrooms cultivated using Korean and Chinese substrates at the same farm in Korea. The results of δ^13^C, δ^15^N, δ^18^O, and δ^34^S differed significantly with the Korean and Chinese substrates (*p* < 0.05), although SIR variations among the samples were mainly found with respect to the harvesting cycle. The maximum differences in the shiitake mushrooms according to the harvesting cycle were <1.0‰ for δ^13^C and δ^15^N, <2.0‰ for δ^18^O, and <3.0‰ for δ^34^S; these values were sufficiently small to discriminate the substrate origin. Only δ^34^S decreased as the harvesting cycle increased, and no clear trends were observed for δ^13^C, δ^15^N, or δ^18^O. Interestingly, shiitake mushroom samples cultivated using the Chinese substrate showed higher δ^13^C, δ^18^O, and δ^34^S compared with those cultivated using the Korean substrate; however, δ^15^N showed the opposite effect, with a higher value in shiitake mushrooms following cultivation using the Korean substrate (*p* < 0.05).

In fact, in the substrates, nutrient sources (C, N, and S) are crucial components for optimal growth and for promoting mushroom growth because mushrooms cannot synthesize the nutrients required for their growth cycle [32]. Additionally, owing to various physical, chemical, and microbial fractionations in nature, the SIRs of bioelements (i.e., C, N, O, and S) can explain the environmental and anthropogenic features of agriculture products or foodstuffs. For example, δ^13^C is highly associated with plant photosynthesis type/efficiency, δ^15^N is related to local agricultural practices, δ^18^O is related to geoclimatic conditions, and δ^34^S is well reflected by geological features [33,34,35]. Therefore, SIR analysis has been widely used for determining the authenticity of agricultural products, including their geographical origin [36,37,38,39]. Accordingly, the δ^13^C, δ^15^N, δ^18^O, and δ^34^S compositions may differ in the substrate used for mushroom cultivation according to the mushroom farm, region, or country and could reflect their isotopic characteristics in the mushroom fruiting body [16,24,40].

According to a previous study [16], δ^13^C, δ^15^N, δ^18^O, and δ^34^S in the dried shiitake samples enable clear discrimination and prediction of the geographical origin (when comparing China and Korea), with a probability of at least 98.7%. The isotopic features of shiitake mushrooms, which showed a higher mean value and wider distribution of δ^34^S, were consistent with those reported in the current study. Interestingly, despite the small sample sized-preliminary results of this study, the mean δ^15^N was higher in the shiitake mushrooms cultivated using the Korean substrate (3.02‰) than in those cultivated using the Chinese substrate (−1.33‰). This trend was similar to that reported previously [41], wherein the mean δ^15^N of the dried shiitake produced via mycelial cultivation from Japan (2.9‰) and Brazil (4.5‰) was higher than that produced from China (0.4‰). Thus, substrates can be a crucial reason underlying the δ^15^N difference in shiitake mushroom. Additionally, another study [24] reported regional and farm-based δ^13^C, δ^15^N, δ^18^O, and δ^34^S differences in *A. bisporus* mushrooms in Korea. Despite the lack of detailed information on the cultivation substrate for *A. bisporus* mushroom, the difference in substrate components or their proportions used in a particular region or farm examined may be considered the most crucial factor in terms of the regional and farm-based SIR variations in the same *A. bisporus* cultivars (Saedo, Saehan).

Our current study also reported the isotopic features and difference between shiitake fruiting bodies produced using two cultivation substrates; however, the SIR data for the cultivation substrates have not been included currently. Therefore, the SIR analysis of cultivation substrates together with more shiitake samples would be necessary to accurately confirm our current findings. This study is the first to report the weak effects of the harvesting cycle on δ^13^C, δ^15^N, δ^18^O, and δ^34^S variations in shiitake mushrooms cultivated using substrates. Moreover, the different origins of the substrates used resulted in variations in FA levels according to the harvesting cycle but showed nonsignificant variations in δ^13^C, δ^15^N, δ^18^O, and δ^34^S. These findings suggest that analyses of δ^13^C, δ^15^N, δ^18^O, and δ^34^S may be more useful than analyses of metabolites, such as FAs, for the identification of the geographical origin labelling of shiitake mushrooms.

## 4. Conclusions

In summary, our preliminary results showed variations and differences in FAs, δ^13^C, δ^15^N, δ^18^O, and δ^34^S in the shiitake mushrooms based on the substrate origin and the harvesting cycle. Eight FAs (C16:0, C17:0, C17:1, C18:0, C18:1 *n*-9, C18:2 *n*-6, C18:3 *n*-6, and C24:0) were found in shiitake mushrooms, and linoleic acid was the most abundant FA, accounting for 77–81% of the total FAs during the four harvesting cycles. The UFA and total FA levels in shiitake mushrooms cultivated using Korean or Chinese substrate continually decreased until the fourth harvesting cycle, whereas SFA levels did not show major variations after the second harvesting cycle. The SIRs (i.e., δ^13^C, δ^15^N, δ^18^O, δ^34^S) differed significantly in the mushrooms cultivated using the Korean and Chinese substrates, and the maximum differences according to the harvesting cycle were <1.0‰ for δ^13^C and δ^15^N, <2.0‰ for δ^18^O, and <3.0‰ for δ^34^S. However, the SIR differences were sufficiently smaller to discriminate the substrate origin compared with differences in shiitake mushrooms grown using the Korean and Chinese substrates. Consequently, our findings verified differences in FAs and SIRs in shiitake mushrooms based on the substrate origin and harvesting cycle. Overall, our findings may provide important insights into recent issues related to the origin labeling of shiitake mushrooms produced in Korea by bag cultivation using Korean or Chinese substrate. However, further study should need to validate more clearly the effect of the cultivation substrates about FAs and SIRs levels along with more shiitake and substrate samples collected from both countries.

## Figures and Tables

**Table 1:** Fatty acid (FA) compositions and levels in shiitake mushrooms based on the harvesting cycle and cultivation substrate origin (mg·g ^−1^ dry weight basis).

Fatty Acid	Harvesting Cycle (H)				Substrate Origin (S)		*p* Value		
	First	Second	Third	Fourth	Chinese	Korean	Main Factor		Interaction
	*n* = 20	*n* = 20	*n* = 20	*n* = 20	*n* = 40	*n* = 40	H	S	H × S
Palmitic acid (C16:0)	2.24 ± 0.26 ^a^	2.00 ± 0.14 ^b^	1.86 ± 0.45 ^bc^	1.71 ± 0.17 ^c^	1.96 ± 0.29	1.95 ± 0.38	****	ns	ns
*cis*-10-Heptadecenoic acid (C17:1)	0.17 ± 0.09	0.18 ± 0.11	0.16 ± 0.12	0.21 ± 0.12	0.19 ± 0.12	0.17 ± 0.10	ns	ns	***
Stearic acid (C18:0)	0.22 ± 0.03 ^a^	0.20 ± 0.03 ^a^	0.18 ± 0.02 ^b^	0.21 ± 0.04 ^a^	0.21 ± 0.03 ^a^	0.19 ± 0.03 ^b^	***	*	ns
Oleic (*cis*) and elaidic (*trans*) acid (C18:1 *n*-9)	0.38 ± 0.04 ^a^	0.30 ± 0.04 ^b^	0.27 ± 0.04 ^c^	0.24 ± 0.86 ^d^	0.31 ± 0.07	0.30 ± 0.06	****	ns	*
Linoleic (*cis*) and linolelaidic (*trans*) acid (C18:2 *n*-6)	13.46 ± 1.71 ^a^	12.08 ± 0.87 ^b^	10.84 ± 0.86 ^c^	8.69 ± 1.72 ^d^	10.82 ± 2.23 ^b^	11.72 ± 2.12 ^a^	****	**	ns
Lignoceric acid (C24:0)	0.24 ± 0.07 ^a^	0.16 ± 0.03 ^b^	0.12 ± 0.03 ^c^	0.15 ± 0.02 ^b^	0.17 ± 0.05	0.17 ± 0.08	****	ns	**
Total (∑FAs detected)	16.71 ± 1.94 ^a^	14.93 ± 1.06 ^b^	13.44 ± 0.96 ^c^	11.22 ± 1.97 ^d^	13.65 ± 2.62 ^b^	14.49 ± 2.41 ^a^	****	*	ns
∑SFA	2.70 ± 0.29 ^a^	2.37 ± 0.17 ^b^	2.16 ± 0.44 ^c^	2.07 ± 0.20 ^c^	2.34 ± 0.34	2.31 ± 0.42	****	ns	ns
∑UFA	14.00 ± 1.68 ^a^	12.57 ± 0.92 ^b^	11.28 ± 0.0.92 ^c^	9.14 ± 1.82 ^d^	11.31 ± 2.31 ^b^	12.19 ± 2.15 ^a^	****	**	ns

Data represent the mean values (±standard deviations) of FAs. ^a–d^ Values with different superscripts differ significantly with respect to the harvesting cycle or substrate origin. ns: not significant, * *p* < 0.05, ** *p* < 0.01, *** *p* < 0.001, **** *p* < 0.0001. ΣSFA: sum of SFAs and ΣUFA: sum of UFAs.
